# Biomarker Analysis of Formalin-Fixed Paraffin-Embedded Clinical Tissues Using Proteomics

**DOI:** 10.3390/biom13010096

**Published:** 2023-01-03

**Authors:** Ekenedirichukwu N. Obi, Daniel A. Tellock, Gabriel J. Thomas, Timothy D. Veenstra

**Affiliations:** School of Pharmacy, Cedarville University, Cedarville, OH 45314, USA

**Keywords:** formalin-fixed paraffin-embedded tissues, mass spectrometry, biomarker discovery, disease diagnosis

## Abstract

The relatively recent developments in mass spectrometry (MS) have provided novel opportunities for this technology to impact modern medicine. One of those opportunities is in biomarker discovery and diagnostics. Key developments in sample preparation have enabled a greater range of clinical samples to be characterized at a deeper level using MS. While most of these developments have focused on blood, tissues have also been an important resource. Fresh tissues, however, are difficult to obtain for research purposes and require significant resources for long-term storage. There are millions of archived formalin-fixed paraffin-embedded (FFPE) tissues within pathology departments worldwide representing every possible tissue type including tumors that are rare or very small. Owing to the chemical technique used to preserve FFPE tissues, they were considered intractable to many newer proteomics techniques and primarily only useful for immunohistochemistry. In the past couple of decades, however, researchers have been able to develop methods to extract proteins from FFPE tissues in a form making them analyzable using state-of-the-art technologies such as MS and protein arrays. This review will discuss the history of these developments and provide examples of how they are currently being used to identify biomarkers and diagnose diseases such as cancer.

## 1. Introduction

Over the past two decades the number of proteins identified in global proteomic studies using liquid chromatography combined with mass spectrometry (LC-MS) has increased from a few hundred to several thousand [1,2]. Even the identification of proteins within serum and plasma, which is notoriously difficult to characterize owing to its wide range of protein concentrations, increased from about 500 to over 4000 proteins [3,4]. This increase catalyzed the rapid increase in the number of studies focused on identifying biomarkers within blood [5]. While the number of studies exponentially increased, the number of clinically validated biomarkers did not. The reasons for this are both technical and physiological. Blood samples are incredibly complex containing proteins that originate from virtually every area of the body through being actively secreted or leaking from dying or diseased cells [6]. While these processes provide blood with a rich diversity of proteins, this diversity is dominated by 22 proteins that make up about 99% of its protein content [3]. This domination makes detecting the lowest abundant proteins, which are anticipated to contain the highest yield of biomarkers, technically challenging owing to the limited dynamic range of analytical instrumentation [7]. While methods such as high abundant protein depletion, chromatographic separation, data-independent analysis, etc. have enabled greater coverage of lower abundant proteins, they cannot overcome the physiological barriers in finding disease-specific biomarkers in blood [8,9,10].

The physiological barriers to identifying biomarkers arise from the ubiquitous nature of the interaction between blood and the body. No cell is more than four cell units removed from the circulatory system and cellular proteins are constantly being dumped into the bloodstream. While a biomarker’s concentration may be significantly elevated at the site from which it is excreted, its concentration will be diluted at the point where the sample is removed from the patient. This dilution may eliminate any quantitative difference in the biomarker’s level between healthy and disease-affected patients. Another barrier is recognition of the biomarker’s origin. While studies analyzing both blood and tissue from patients to correlate biomarker measurements in both types of samples [11] may help, they do not absolutely prove the source of the biomarker.

## 2. Blood versus Tissue

Identifying a diagnostic biomarker would be helpful regardless of its source, however, if it was measurable from an easily accessed biofluid such as blood, it would be invaluable. This accessibility has made plasma and serum the sample of choice for the discovery of novel biomarkers. Even with the use of advanced technologies the discovery of novel blood-based biomarkers has achieved little success [12]. This failure made scientists realize that bridging the gap between discovery and clinical utility is more challenging than anticipated. Indeed, a huge discrepancy between the number of FDA approved protein biomarkers and the effort put towards this aim continues to exist, with most of the clinically useful circulating biomarkers having been discovered 40 to 60 years ago [13].

This inefficiency caused many investigators to abandon blood-based searches and focus on first identifying the biomarker at the tissue level [14,15,16]. Requiring invasive techniques makes acquiring enough fresh or frozen tissue samples for a proper proteomic study difficult to obtain from human subjects. Since they require refrigeration, these samples are also relatively expensive to store and difficult to transport between institutions. Formalin-fixed paraffin-embedded (FFPE) tissues represent a cost-effective, standardized alternative to long-term storage of frozen tissues. Due to the covalent cross-linking of proteins within these samples, FFPE tissues are stable and can be stored for decades without concern of decay [17]. This stability reduces the cost of storage since these tissues can be stored at room temperature while eliminating many of the challenges in shipping samples between labs around the world. Throughout the decades, enormous libraries containing clinical tissues have been accumulated. For example, Sweden has routinely stored virtually all human tissues acquired from surgical procedures in FFPE form for decades [18]. While the use of FFPE tissue in the field of proteomics has been historically linked to immunohistochemistry (IHC), this trend began to change in the early part of this century.

## 3. The Development of FFPE Tissue Proteomics

Between 2005–2007, two articles were published that demonstrated that hundreds of proteins could be identified from FFPE tissues using MS [19,20]. Prostate biopsy samples were used in both studies as well as shotgun proteomic methods in which proteins extracted from the tissues are digested with trypsin and analyzed using LC-MS. Both studies compared normal, benign prostatic hyperplasia (BPH), and prostate cancer (PCa) cells for changes in protein abundance. One study was able to identify 428 proteins expressed within the prostate samples [19], which is an impressive number considering the exploratory nature of the study and the state of LC-MS at the time it was conducted. By adding a standard to the sample prior to LC-MS analysis, this group was able to confirm the upregulation of Wnt-3 in cancerous cells (in agreement with IHC analysis).

The study completed in 2005 also examined prostate tissue and incorporated laser capture microdissection (LCM) to extract approximately 200,000 cells from cancerous and BPH regions of the FFPE tissue sections [20]. Tryptic peptides corresponding to 702 and 1156 proteins from BPH and PCa tissue extract were identified, respectively. Quantitative analysis of the data set was performed using a subtractive approach in which the number of peptides identified in each sample correlates with the protein’s abundance. Using this approach, prostate-specific antigen (PSA) and macrophage inhibitory cytokine I (MIC-1) were shown to be of higher abundance in PCa cells compared to BPH cells.

This study also compared the number of proteins that could be identified from equivalent fresh frozen and FFPE tissues. A mouse liver tissue was cut in half and one section was frozen and embedded in optimal cutting temperature (OCT) compound while the other was FFPE. Tissue sections were cut from the faces of each and approximately 30,000 cells retrieved using LCM. Just over 2000 peptides corresponding to 776 unique proteins were identified from the frozen tissue section, while just over 1700 peptides and 684 unique proteins were identified in the FFPE tissue. There was significant overlap in both the identity and number of peptides identified for the top 15 proteins seen in both samples (Table 1). For example, carbamoyl phosphate synthase was the most readily identified protein in both samples having been identified by 91 and 72 unique peptides in the frozen and FFPE tissues, respectively. This finding, along with many others, showed that the information content obtained from FFPE and frozen tissue was similar [21,22,23,24,25].

## 4. Protein Extraction Methods from FFPE Tissue

In retrospect, it is somewhat puzzling why it took so many years for FFPE tissues to become a commonly used sample in MS-based proteomic analysis. In our opinion it was related to two reasons: (1) many proteomic laboratories were unaware of the vast archive of tissue that was available and (2) it was thought that the formalin-fixation made it impossible to retrieve analyzable protein. In the end, most MS-based proteomic studies used extraction methods based on antigen retrieval (AR) methods originally developed for IHC, which simply use a combination of buffer and heat [26]. To enhance the recovery of proteins for MS analysis, researchers have used various combinations of heat and buffers that contain detergents and/or reducing agents such as dithiothreitol or glycine [27,28,29,30]. As the popularity of FFPE tissue in proteomic analysis grew several companies including Qiagen, Hilder, Germany, Agilent Technologies, Santa Clara, CA, USA, Invent Biotechnologies, Inc., Plymouth, MN, USA, Covaris, Woburn, MA, USA and Bio Basic, Markham, ON, USA develop kits that could be used to help simplify and standardize protein extraction.

One concern when using FFPE tissues is to what extent are formalin crosslinks reversed during sample preparation. This question is generally answered through the direct comparison of matching FFPE and fresh frozen tissues. As described above, most sample preparation methods use a combination of buffer, detergent, reducing agents, and heat, with some also using elevated pressure. Studies comparing matched FFPE and fresh frozen tissue have generally shown that these conditions are sufficient to reverse a majority of protein crosslinks. For example, a study by Addis et al. [31] extracted proteins from FFPE and fresh frozen sheep tissues by immersing tissue sections in buffer comprised of 20 mM Tris HCl, 2% SDS, and 200 mM DTT (pH 8.8) and heating the sample at 100 °C for 20 min, followed by additional heating at 80 °C for 2 h. Comparison of the samples using SDS-PAGE, Western blotting, and LC-MS showed similar results suggesting that the reversal of the crosslinks does not impact the ability to identify peptides obtained from the intact proteins. Gene ontology analysis of the identified proteins showed no significant bias in the proteins identified in the FFPE or fresh frozen tissues.

Another study extracted proteins from matching FFPE and fresh frozen mouse livers using 0.1 M Tris HCL, 10 mM sodium deoxycholate, and 10 mM sodium lauroyl sarcosinate (pH 9.0) and heated the samples at 95 °C for 60 min [32]. The samples were also subjected to 60 cycles at 45,000 psi for 95 s, followed by 5 s at atmospheric pressure. Finally, the samples were subjected to 50 cycles at 45,000 psi for 20 s and 15 s at atmospheric pressure. This study showed that the addition of pressure cycling technology (PCT) to the sample preparation more than tripled the amount of protein extracted from the FFPE tissue, increasing the extraction efficiency to almost 100%.

So how do these combinations of buffers, detergents, heat, and pressure reverse formalin crosslinks? The key is hydration. The prevailing hypothesis is that heating the FFPE tissue in the presence of a buffer and detergent denatures that protein molecules and allows water molecules to access cavities that will hydrolyze the formalin-protein bond [33]. This hypothesis is consistent with the observation that heat treatment combined with high pressure increases the extent of the reversal of formalin-protein crosslinks by increasing the level of hydration within the protein’s interior [34,35,36].

In the early days of developing methods for extracting proteins from FFPE tissues for proteomic analysis, Fowler et al. (2007) performed a series of studies to evaluate the use of different buffers, detergents, reducing agents, and temperatures on the extraction efficiency [37]. In these studies, they prepared a FFPE surrogate tissue using a known concentration of lysozyme, ribonuclease A or a 1:2 molar ratio of carbonic anhydrase and lysozyme. The study showed that heat, a denaturant, and a detergent were all necessary for optimal protein extraction. Sodium dodecyl sulfate (SDS) was the single most important ingredient for protein extraction. A buffer containing 2% SDS had a protein extraction efficiency 13-fold greater than buffers without SDS. While Triton X-100 is an often-used protein detergent, it was not very effective on extracting FFPE proteins. Heating the tissues at either 80 °C for 2 h or 100 °C for 20 min was the most effective for extracting proteins. The addition of reducing agents such as glycine showed a modest benefit in increasing protein extraction. The optimal pH range was found to be between 4–6, however, results using model proteins of various isoelectric points (pI) suggested that extraction of any individual protein was dependent on the pH and pI of the specific protein.

**Figure 1:**
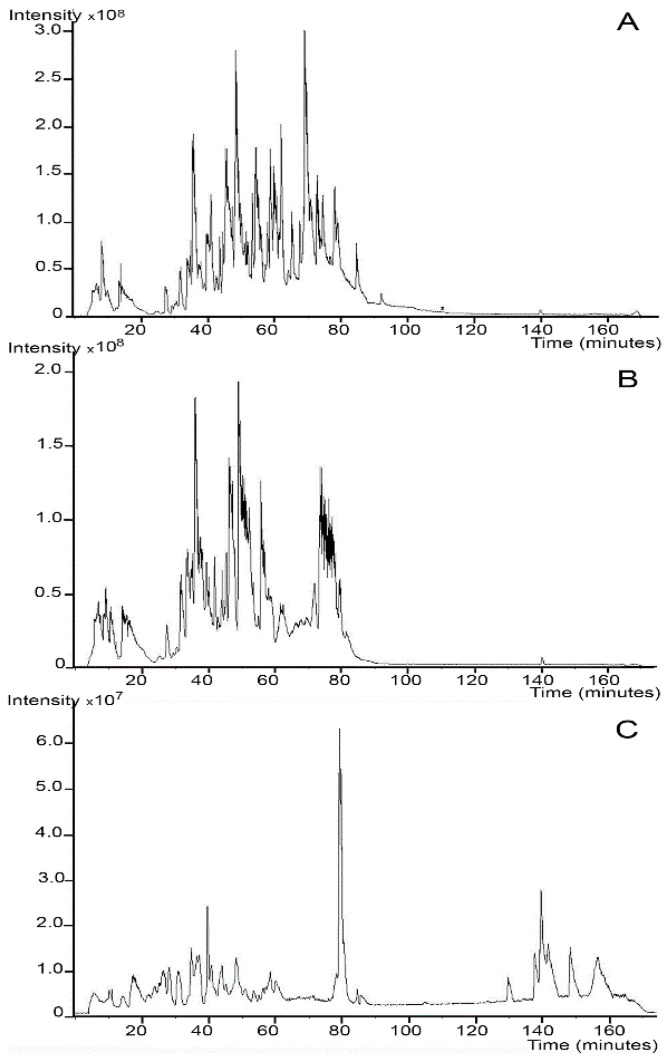
Comparison of mass spectrometry (MS) profiles (i.e., extracted ion chromatograms) of native protein mixture and tissue surrogate extracts. Formalin-fixed paraffin-embedded (FFPE) tissues were heated in 50 mM Tris, pH 8 and 2% SDS at either elevated (40,000 psi) or atmospheric (14.7 psi) pressure. The extracts were analyzed using liquid chromatography (LC)/MS. The MS profiles of each FFPE extract were compared to the native, unfixed protein mixture. (**A**) native, unfixed tissue surrogate mixture; (**B**) FFPE tissue surrogate extracted at 40,000 psi; (**C**) FFPE tissue surrogate extracted at 14.7 psi, adapted with permission from ref. [39].

This same group was also instrumental in showing the value of pressure in extracting proteins from FFPE tissues [38,39]. Using a FFPE lysozyme sample, they extracted the protein at pressures ranging from 14.7 to 50,000 psi. The results showed that extraction of the FFPE sample using a buffer and SDS at 40,000 psi increased the percentage of protein extracted from the lysozyme sample from 96% from only 26% observed when the extraction was performed at atmospheric pressure (i.e., 14.7 psi). The effect of using higher pressure for extraction on the LC-MS profiles (extracted ion chromatograms and protein identifications) was also evaluated [39]. Proteins were extracted from a multi-protein FFPE tissue surrogates in Tris-HCl buffer with 2% (*w*/*v*) SDS at both 40,000 and 14.7 psi, digested with trypsin, and analyzed using LC/MS. At 14.7 psi and pH4, only lysozyme and RNase A were identified using MS/MS, while none of the component proteins were correctly identified at pH 8. Increasing the extraction pressure to 40,000 psi resulted in the identification of all five surrogate proteins at both pH 4 and 8, with sequence coverages ranging from 28% to 69%. These results were comparable to those obtained when the surrogate protein mixture was analyzed prior to fixation. Besides enhanced peptide identification, the false identification rates for the pressure extracted samples were only 5.7% (pH 8) and 7.8% (pH 4), compared to the rates for the non-pressure extracted tissue surrogates of 42% (pH 4) and 100% (pH 8). The MS spectra of the native protein mixture, pressure-extracted, and non-pressure extracted multi-protein surrogate samples showed differences in protein quality (Figure 1). The unfixed protein mixture spectrum (panel A) showed several well resolved peaks eluting as did the profile for the tissue surrogate extracted under elevated pressure (panel B). Many of the peaks within the spectrum of the non-pressure treated FFPE surrogate mixture (panel C) were reduced in intensity and eluted later, which suggests that much of the protein material remained cross-linked and was not completely digested.

## 5. The Movement to Global Analysis

As the realization that FFPE tissues were a viable option for proteomic analysis grew, more laboratories began analyzing these samples. With increased interest came an escalation in the scale of the biomarker discovery studies being conducted using FFPE tissues. The numbers of quantifiable proteins identified from FFPE tissues quickly grew from the hundreds to the thousands [40,41]. The types of different cancers analyzed through FFPE tissues also expanded to include a diverse group such as ovarian and hepatocellular [42,43,44]. Shortly after, studies describing the identification of post-translational modifications (PTMs), especially sites of phosphorylation and glycosylation, began to arise [45,46]. As of December 2022, there are 432 articles listed on PubMed under the search terms “FFPE” and “mass spectrometry”. This number is impressive considering that there were only 52 as of the end of 2010. Table 2 provides a list of just a few of these examples. This list shows how investigators have continued comparing methods for extracting proteins from FFPE tissues, while using existing methods to address basic research such as discovering diagnostic and prognostic biomarkers for specific disease states, characterizing cellular proteomes, etc. While oncology research was and continues to be a major focus, proteomic studies of FFPE tissues began to address other disease areas including neurological disorders, heart disease, and transplant injury.

**Figure 2:**
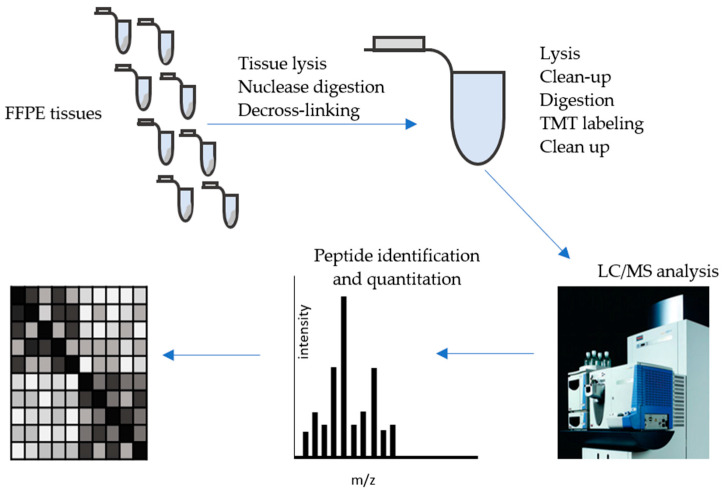
Proteomic analysis of formalin-fixed paraffin-embedded (FFPE) tissue sections using Single-Pot Solid-Phased-enhanced Sample Preparation Clinical Tissue Proteomics (SP3-CTP). In the SP3-CTP method, FFPE tissue sections are lysed, digested with nuclease and the proteins decross-linked. The protein lysate is then digested into peptides, which go through a series of clean up steps along with tandem mass tag (TMT) labeling to generate quantifiable peptides. The peptide lysate is analyzed using LC/MS and the relative abundance of peptides in comparative samples measured. The various tissue samples are segregated based on the relative abundance of a select percentage (e.g., 25%) of proteins that showed the greatest differences in abundance between the samples.

As FFPE became an increasingly popular choice of proteomic sample, it became necessary to develop streamlined methods that incorporated methods to accurately compare the quantities of proteins between various tissue types. A recently developed high-throughput method termed SP3-CTP (Single-Pot Solid-Phased-enhanced Sample Preparation Clinical Tissue Proteomics) has shown the ability to quantitatively compare proteins between hundreds of FFPE tissues, as summarized in Figure 2 [41,59]. In the SP3-CTP method, deparaffinized tissue sections are lysed using a combination of enzymatic treatment, detergents, and heat. Following reduction and alkylation, the concentration of the extracted proteins is measured using a BCA assay. Magnetic beads with hydrophobic and hydrophilic surfaces were added to protein mixture, along with an aliquot of an *E. coli* lysate to monitor sample variability. After incubation, proteins bound to the magnetic beads were extracted and the supernatant discarded. The beads were rinsed with washes of 70% ethanol followed by acetonitrile. The beads were resuspended in aqueous buffer and digested with a combination of trypsin and Lys-C enzymes. Peptides were separated from the magnetic beads using sonication, at which point the magnetic beads were discarded. Extracted proteins are enzymatically digested with a combination trypsin/Lys-C cocktail and the resulting peptides isolated and quantitated. Equal amounts of peptides from each sample are labeled using tandem mass tag (TMT) labels, which incorporate stable-isotope labeled tags that enable the quantity of peptides in different samples to be compared [60]. After a series of solvent exchanges, desalting, and concentration steps, the labeled peptides are analyzed using LC-MS.

An application of SP3-CTP profiled the proteomes of 300 archived FFPE breast tumor primary tissues along with 38 normal reduction mammoplasty tissues acquired from patients diagnosed in two separate time periods (2008–2013 and 1986–1992) [61]. The aim of the study was to develop a more accurate classification system enabling tumors to be diagnosed and treated with greater certainty. While DNA and RNA profiling has been used in the past, these classifications are not always useful in guiding therapies since tumors have extensive heterogeneity beyond their genetic profiles. In this study, the FFPE breast tumor samples were segregated using the five PAM50 breast cancer subtypes, which classify samples based on a 50-gene signature (i.e., luminal A, luminal B, Her2-enriched, basal-like, and normal-like) [62]. Peptides were extracted from 3–6 FFPE sections per sample, with each section comprised of at least 80% tumor cells. The samples were processed using the SP3-CTP method, and the peptides extracted from the tumor tissues were quantitatively compared. A total of 9088 proteins were quantified within the study with an impressive 4214 of these being quantified across all samples. In addition, 706 synthetic peptides corresponding to 179 biologically important, but low abundant, proteins were added to each sample to increase the chance each would be quantitated in the MS assays.

An unsupervised clustering algorithm was applied to the 25% most highly variable proteins found across all samples that passed quality control. The analysis produced four clusters as shown in Table 3. Each cluster contained a unique set of proteins that differentiated them. Cluster 1 contained tumors with an increased level of proteins involved in fatty acid metabolism, catabolic, and oxidation/reduction processes. Enriched stromal and extracellular matrix (ECM) processes (e.g., collagen organization, blood coagulation, and angiogenesis) were characteristic of cluster 2. A high abundance of immune-response related proteins (e.g., MHC class I and II, antigen presentation, immunoproteasome, etc.) were observed within cluster 3. Cluster 4 was enriched for ECM, stromal, blood coagulation, humoral immune response, and hormone receptor binding proteins, but deficient in proteins related to DNA damage repair.

The proteomic data acquired for the 88 triple negative breast cancer (TNBC) cases as determined using IHC was analyzed using an unsupervised clustering classification focusing on the 25% most highly variable proteins. These cancer cases are of distinct importance in oncology as TNBC is highly invasive and is characterized by its poor prognosis relative to other forms of breast cancer. At least one-third of TNBC patients will show recurrence or distant metastisis [63]. The 88 TNBC cases were categorized as basal-like (61), Her2-enriched (22), and luminal B (5) using PAM50 criteria. Four clusters were identified using the SP3-CTP data: TNBC-Cluster 1 characterized by immune-response, antigen presentation, and type I and II interferon signaling pathways; TNBC-Cluster 2 enriched for ECM, blood coagulation, and humoral immune-response proteins; TNBC-Cluster 3 characterized by high levels of proteins involved in lipid metabolism, as well as catabolic and oxidation/reduction pathways; and TNBC-Cluster 4 that contained higher levels of DNA replication and cell cylce proteins along with some immune-related peptides. While TNBC-Clusters 1, 2 and 4 were mainly basal-like cases, cluster 3 was comprised of mostly Her2-enriched cases. TNBC-Cluster 1 had the most favorable OS, while Cluster 4 had the poorest OS. Comparison of these proteomic-based clusters with those formed using the RNA-based PAM50 classifier showed excellent overlap as each cluster’s character (i.e., basal-immune suppressed, basal-immuned activated, luminal, and mesenchymal) was similar in both classification schemes.

As shown in Table 3, cluster 3 and 2 had the most and least favorable recurrence free (RFS) and overall survival (OS) rates, respectively. Both of these clusters were primarily treated with chemotherapy, while clusters 1 and 4 were treated using hormonal therapies.

While classifying tumors based on proteomic profiles is useful, what truly impacts the current state of diagnostic capabilities is the identification of biomarkers that could be used to quickly predict outcome and direct treatment options. The tumors that had the highest RFS and OS rates were those that were enriched for immune-related proteins (i.e., cluster 3 and TNBC-cluster 1). These results suggest that these tumors may produce an effective anti-tumor immune response [61]. Some of the specific proteins identified in these clusters (e.g., TAP1 and HLA-DQA1) could be incorporated into IHC analyses to direct the oncologist to treat these tumor types with immune-modulating chemotherapies and drugs that block immune checkpoints. Conversely, cluster 2 and TNBC-cluster 4 that had the best RFS and OS rates, showed high levels of fibronectin, could benefit from treatment with angiogenic inhibitors and immune-boosting therapies. Incorporation of some of the biomarkers identified in this study into IHC tests would enable a more accurate classification of the tumor and guide the selection of the most effective therapeutic regimen.

## 6. Targeted, Quantitative Biomarker Analysis

Analysis of FFPE tissues using IHC was first demonstrated by Shi et al. in 1991 [64] and along with hematoxylin-eosin staining, is still considered the gold standard for classifying tissues. Unfortunately, IHC analysis of FFPE tissues has several issues that can limit its effectiveness including variability in tissue preparation, poor antibody specificity, low throughput, lack of scoring standardization. While IHC data is critical for making diagnostic or therapeutic decisions, its quantitation is limited by low resolution. The colorimetric signal produced by IHC is used to generate a score based on the number of cells attaining a certain staining intensity that is rated from 0 (no staining) to 3 (intense staining). Even with the development of imaging and signal quantitation software and hardware platforms, quantitation remains subjective for characterizing biomarkers in cancer patient tissues. With the proven ability to extract proteins from FFPE tissue and analyze them using MS, investigators began turning to targeted MS methods to quantitate the absolute abundance of biomarkers more accurately in these tissues.

**Figure 3:**
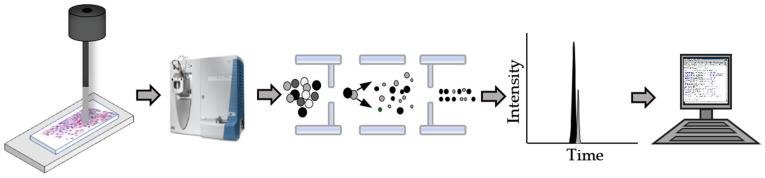
Quantitation of biomarkers extracted from formalin-fixed paraffin-embedded (FFPE) tissue using Liquid Tissue-selected reaction monitoring mass spectrometry (SRM-MS). Specific areas are removed from the FFPE tissue using laser capture microdissection. These sections are placed in a tube to be deparaffinized and the proteins extracted and digested into peptides. At this stage a known amount of stable isotope labeled internal standard is added to enable the absolute quantitation of specific biomarkers to be measured using SRM-MS. The patient-specific data is then analyzed to diagnose the tumor or determine the best course of treatment.

One of the first examples analyzed the hepatocyte growth factor receptor (*MET*), a known driver of various cancers including lung, gastroesophageal, ovarian, and renal [65]. Its measurement in FFPE tissues using IHC is routinely used as a diagnostic and prognostic indicator. To increase the accuracy of *MET* measurements in tumors, a Liquid-Tissue-SRM (selected reaction monitoring) MS method was developed [66]. In this method, tumor cells are isolated from FFPE tumor tissues using laser microdissection. A cell lysate is prepared from these cells and digested into peptides using trypsin. To measure the absolute quantity of the *MET* protein, a known amount of a stable isotopically labeled peptide corresponding to a unique peptide in *MET*, is added to the digested lysate. The complete mixture is analyzed using LC-MS with the mass spectrometer set up in SRM mode so that the specific *MET* peptide and its isotope labeled internal standard are automatically selected for analysis. The absolute amount of *MET* within each tumor is accurately measured by comparing the sample result to calibration curves (Figure 3). The results are then used to diagnose the tumor and determine the most efficacious therapy.

In this application of the Liquid-Tissue SRM-MS method, 130 gastroesophageal (GE) FFPE tumor tissues were analyzed using Liquid-Tissue SRM-MS, IHC, mean *MET* gene copy number/nucleus, and mean *MET*/*CEP7* (centromere 7) gene copy number. While the *MET*/nucleus and *MET*/*CEP7* gene copy results showed high correlation with the Liquid-Tissue SRM-MS results (R^2^ = 0.898), surprisingly the correlation between the SRM-MS and IHC results was low (R^2^ = 0.537). This low correlation suggests that the subjectivity and variability associated with IHC may not provide the most accurate assessment of *MET* status in these tissues.

A similar study using multiple reaction monitoring (MRM)-MS was used to quantitate Her2 in 210 FFPE breast tumors [67]. The samples were processed using the Liquid-Tissue method and the absolute abundances of both Her2 and adhesion molecule A were measured using a combination of stable-isotope labeled internal standards and MRM-MS. Adhesion molecule A levels were measured as a way of normalizing the Her2 levels between tumor samples as the abundance of adhesion molecule A should remain constant. The Her2 gene copy number was also measured using fluorescence in situ hybridization (FISH) to determine if there was any gene duplication events that occurred within the tumor cells. FISH testing is generally done on Her2 tumors assigned an IHC score of 2+ or 3+. Approximately 80% of invasive breast cancers are tested for Her2 using IHC and only 20% are tested using FISH. Both the American Society of Clinical Oncology and College of American Pathologists recommend treating FISH-positive Her2+ tumors with an IHC score of +2 with trastuzamab but using standard chemotherapy for patients whose tumors are FISH-negative or have IHC scores of 0 or 1+. In this study of 210 FFPE tissues, the MRM-MS results were able to correctly distinguish Her2 2+/FISH positive tumors from Her2 2+/FISH negative tumors. In contrast, IHC could not distinguish the FISH status of Her2 2+ tumors. Being able to conclusively categorize the Her2 expression level and the FISH status of the tumor cells suggests that MRM-MS provides a more accurate measure by which to decide on the correct therapy for treating the patient.

There are many advantages to using the Liquid Tissue-SRM method compared to standard IHC. The Liquid Tissue-SRM method does not require antibodies or other types of affinity reagents. It can be scaled to quantitate several protein biomarkers from small amounts of tissue samples, making it extremely economical when clinical tissue samples are scarce. For example, a recent study demonstrated the ability to measure the absolute abundance of 200 specific proteins extracted from FFPE breast tumors [68]. The Liquid-Tissue results are completely objective as they do not require a pathologist to judge the number and staining intensity of individual cells. Removal of this subjective factor makes the technique universally applicable to clinical labs worldwide if a standardized protocol is followed.

## 7. Challenges

While the analysis of FFPE tissues in the search and analysis of proteomic biomarkers is becoming increasingly common, there are still some uncertainties how representative the proteins extracted from these tissues are related to actual tissue proteome. There are several technical issues related to the acquisition and preservation of the FFPE tissues that can affect the proteomic results. There are no standardized protocols for preparing FFPE tissues for proteomic analysis and none existed in the past. While the methods used for preserving the tissues between the time of resection and fixation can affect their proteomes, they have not been standardized for proteomic studies. Most variability is introduced by the fixation procedure, primarily the thickness of the tissue, the amount of fixative used, and fixation time [69]. A study by Tanca et al. showed that the ability to extract and identify peptides from FFPE tissues is inversely proportional to the fixation time [70]. Therefore, standardizing fixation time and standardizing the formalin:tissue volume ratio and fixation time would appear to be a critical needs as specimens are often fixed over vague time periods such as overnight or over the weekend [71]. Quite interestingly is the fact that many studies have shown that storage time has little effect on the proteome results obtained from FFPE tissues [72,73,74], although it could affect the protein extraction efficiency.

Accurate identification of peptides retrieved from FFPE tissues is another challenge. A vast majority of studies only search for unmodified tryptic peptides, however, formalin-fixation is known to produce a wide variety of covalently modified amino acids. A study using a model protein (i.e., insulin) to identify formalin-induced modifications confirmed the high reactivity of Arg, Lys, and Tyr residues but failed to show any detectable modification of eight other reactive amino acids [75]. Other modifications due to methylolation (+30 Da on Asn, Cys, His, Lys, Tryp, Tyr, and N-terminus residues), imination (+12 Da on Lys, Trp, and the N-terminus residues, formaldehyde-glycine adducts (+99, +198, +87, +174 Da on Arg, Asn, Glu, His, Trp, Tyr, and the N-terminus residues), and methylene bridges (+12) resulting from the fixation method were observed. Another study using histones identified a series of methylated, demethylated, acetylated, and ubiquitinated residues, while others have provided putative identification of up to 24 other types of modifications within proteins extracted from FFPE tissue [76]. Unfortunately, none of these putative identifications were independently validated. Present technological barriers make searching the entire modification space too exhaustive and time consuming. Yet, it is critically important to know the stoichiometry of any peptide modification as it is required to assess how reliable a peptide’s abundance can serve as a valid biomarker.

While many factors related to the processing of the FFPE tissues can be controlled once it is in the hands of the proteomics lab, one major factor cannot: the time between resection of the tissue and its fixation in formalin. This parameter is separate from the fixation time, which describes how long the tissue is kept in formalin. In cases where tissues are surgically removed, the length of time a tissue may be subjected to anoxia is dependent on factors such as anesthesia, surgical techniques, and time to resection [77]. The amount of time that a tissue is without oxygen will undoubtedly affect its proteome. While these variables are extremely difficult to control, a system to record this time could be implemented as part of the tissue record [78].

## 8. Discussion

Proteomics, much like genomics, has come a very long way in a relatively short period of time. Twenty years ago, the focus was simply identifying as many proteins as possible in a complex biological mixture, however, thoughts of how proteomics could impact clinical labs were always present. While the path to clinical application was not always evident, we are finally seeing this goal come to fruition. The key was proteomic scientists and clinicians became more aware of the needs and capabilities of each field. This awareness has led to greater collaboration so that proteomic scientists increased their focus on discovering biomarkers that could diagnose cancer at earlier stages or classify tumor types more accurately. Part of this focus entailed developing technologies that could more readily analyze samples that were already being routinely used in the clinic.

Serum and plasma samples dominated the early days of proteomic biomarker discovery. The discovery of a circulating biomarker that could diagnose cancer at an early stage would be invaluable; however, the analytical and physiological challenges were great. While serum/plasma proteomics continued, proteomic scientists became aware of FFPE tissues, which are routinely used for clinical evaluation of tumors but were thought to be intractable to MS-based analyses. In less than 20 years, proteomics has developed methods to more accurately classify tumors based on quantitating specific biomarkers in FFPE tissues enabling greater confidence in the selection of the best therapeutic strategy [41]. Proteomic scientists have also moved forward in developing targeted methods that can quantitate commonly used cancer biomarkers with greater accuracy than IHC, also enhancing the ability to select the proper course of treatment [79]. Like any effective science, technologies and applications continue to make progress. The goal is that someday biomarkers that diagnose cancers at the earliest possible stage and allow for the best therapeutic course to be selected are identified so that the pain and suffering afflicted by cancer in so many people can be alleviated.

## 9. Conclusions

While thought to be intractable to proteomics research, FFPE tissues have become an invaluable resource in the identification of cancer biomarkers. Probably their most valuable characteristic is their sheer number, as pathology departments around the world contain millions of FFPE tissues. Not only are FFPE tissue samples available for every possible tumor (even those that are rare), but they are also easy and inexpensive to store. Another useful factor not discussed in this article is the ability to extract DNA and RNA from these tissues enabling studies that can characterize tumors at the genomic, transcriptomic, and proteomic levels. Accessing information at these levels will enable oncologists to have a greater understanding of cancer. As history has shown, the more we know about how a disease affects the body, the better we are at developing treatments to combat it.

## Figures and Tables

**Table 1:** Comparison of number of tryptic peptides identified in 10 major proteins identified from frozen and formalin-fixed paraffin-embedded (FFPE) mouse liver tissue. In this study, peptides were extracted from a FFPE and fresh frozen section cut from opposite faces of a mouse liver tissue and identified using mass spectrometry.

	Number of Peptides Identified	
Protein	Frozen Tissue	FFPE Tissue
Carbamoyl-phosphate synthase	91	72
78 kDa Glucose-regulated protein	21	33
ATP synthase β chain	34	32
60 kDa Heat shock protein	33	32
10-Formyltetrahydrofolate dehydrogenase	41	32
Catalase	12	30
HMG-CoA synthase	23	29
Acetyl-CoA acyltransferase	27	27
Glutathione-S-transferase	21	25
Pyruvate carboxylase	21	24

**Table 2:** Partial list of studies that have analyzed formalin-fixed paraffin-embedded (FFPE) tissues along with tissue type, purpose of study, extraction conditions, and analysis method. Abbreviations: BPH: benign prostatic hyperplasia; RP-HPLC: reversed-phase high-performance liquid chromatography; ACN: acetonitrile; NH_4_HCO_3_: ammonium bicarbonate; SDS: sodium dodecyl sulfate; cIEF: capillary isoelectric focusing; DTE: 1,4-dithioerythritol; DTT: dithiothreitol; Na_2_EDTA: sodium ethylenediaminetetraacetic acid; SWATH: sequential window acquisition of all theoretical fragment ion spectra; TCEP: tris(2-carboxyethyl)phosphine; CHAPS: 3-[(3-cholamidopropyl)dimethylammonio]-1-propanesulfonate; SDC: sodium deoxycholate; HNSCC: head and neck squamous cell carcinoma; AD: Alzheimer’s disease.

Tissue Type	Purpose	Extraction Conditions	Analysis Method	Ref
Prostate cancer and BPH	Method development	Liquid-Tissue ID and Liquid-Tissue MS (proprietary)	RP-HPLC ion trap	[20]
Prostate cancer	Method development	Samples suspended in 30% ACN, 100 mM NH_4_HCO_3_ buffer and boiled for 10 min.	RP-HPLC ion trap	[19]
Prostate cancer	Biomarker discovery	Homogenized in 4% SDS, 100 mM DTE, 100 mM TrisHCl pH 7.6. Sonicated 3 times for 10 s and 1 h of heating at 90 °C.	RP-HPLC ion trap	[47]
Glioblastoma	Proteome characterization	8 M urea and 20 mM Tris-HCl at pH 8.0	cIEF/RP-HPLC ion trap	[48]
Mouse Liver	Method development	1. 40 mM Tris pH 8.2/6 M guanidine-HCl/65 mM DTT, centrifugation 25,000× *g* 1 h. 2. 40 mM Tris pH 8.2/2% SDS, 100 °C 20 min, 60 °C 2 h. 3. 40 mM Tris pH 8.2/6 M guanidine-HCl/65 mM DTT. 4. 40 mM Tris pH 8.2/6 M guanidine-HCl/ 65 mM DTT, 100 °C 30 min.	RP-HPLC ion trap	[49]
Prostate cancer and diffuse large B-cell lymphoma	Tumor stratification	6 M Urea, 2 M thiourea, 5 mM Na_2_EDTA in 100 mM NH_4_HCO_3_, pH 8.8. Lysed using pressure cycling at 45,000 psi.	RP-HPLC-SWATH	[50]
Adrenal cortical carcinoma	Biomarker discovery	4% SDS, 1 mM TCEP, and 0.3 M Tris pH 8.0. After sonication, the samples were incubated at 95 °C.	RP-HPLC-Orbitrap	[51]
Kidney	Renal allograft injury biomarkers	20 mm Tris, 2% SDS, pH 8.0. Sheared using 18- and 23-gauge needles followed by ultrasonication and heating at 98 °C.	RP-HPLC-Orbitrap	[52]
Colorectal cancer	Methods comparison	FASP Kit (proprietary; Expedeon) vs. 10 mM NH_4_HCO_3_, pH 6.0 and heated at 95 °C for 1 h.	UPLC-qTOF	[53]
Testicular tissue	Azoospermia subtypes biomarkers	4% SDS, 5 mM MgCl_2_x6H_2_O, 10 mM CHAPS, 100 mM NH_4_HCO_3_, 0.5 M DTT. Followed by sonication and incubation at 95 °C.	RP-HPLC qTOF with ion mobility	[54]
HNSCC	Diagnostic and prognostic biomarkers of HNSCC	Liquid Tissue (proprietary)	RP-HPLC Ion Trap	[55]
Breast cancer	Biomarkers	Liquid Tissue (proprietary)	RP-HPLC Ion Trap	[56]
Temporal cortex neurons	AD-associated proteins	20 mM DTT at 57 °C for 1 h followed by 50 mM at room temperature. Method performed with and without RapiGest surfactant	RP-HPLC-Q-Exactive	[57]
Substantia nigra	Proteome characterization	2% SDS in 300 mM Tris-HCl pH 8.0, or 1% SDC in 300 mM Tris-HCl pH 8.5 or 0.2% Rapigest (proprietary) in 50 mM NH_4_HCO_3_. Heated at 99 °C and sonicated.	RP-HPLC-Q-Exactive	[58]

**Table 3:** Characteristics of four proteome-based clusters found with proteins quantitated in formalin-fixed paraffin-embedded samples obtained from patients diagnosed with breast cancer between 2008–2013 and 1986–1992 [43]. While 9088 proteins were quantified in total, an impressive 4214 proteins were quantified in every sample. RFS: recurrence free survival in years; OS: overall survival.

Cluster	Number of Samples	RFS/OS Rank	Characteristics
1	34	2	Luminal B (n = 18) Her2-enriched (n = 13)
2	50	4	Basal-like (n = 41) Her2-enriched (few)
3	47	1	Basal-like (n = 31) Her2-enriched (n = 14)
4	43	3	Her2-enriched (n = 26) Luminal A (n = 8) Luminal B (n = 8)
		

## Data Availability

Not applicable.
